# Fracture Nonunions and Delayed Unions Treated With Low-Intensity Pulsed Ultrasound Therapy: A Clinical Series

**DOI:** 10.7759/cureus.17067

**Published:** 2021-08-10

**Authors:** Kavyansh Bhan, Ronak Patel, Kamrul Hasan, Mahesh Pimplé, Sucheta Sharma, Varsha Nandwana, Mhafrin Basta

**Affiliations:** 1 Trauma & Orthopaedics, Barts Health NHS Trust, London, GBR; 2 Orthopaedic Surgery, Punjab Institute of Medical Sciences, Jalandhar, IND; 3 Orthopaedic Surgery, Lady Hardinge Medical College, Delhi, IND; 4 Dermatology, James Cook University Hospital, Middlesbrough, GBR

**Keywords:** nonunions, delayed union, lipus therapy, ultrasound therapy, radiological and clinical fracture healing

## Abstract

The incidence of nonunion of fractures has been steadily rising owing to improved life expectancy following severe injuries along with rising cases of polytrauma. Once a nonunion is established, the chances of spontaneous healing are deemed to be quite low. Fracture nonunion continues to be a challenge in clinical practice with nonunions having a considerable impact on patient’s quality of life causing both functional and psychosocial disability. Low-Intensity Pulsed Ultrasound (LIPUS) therapy is being projected as a viable and non-interventional alternative to surgical management of nonunions and delayed unions. LIPUS therapy is being widely recommended as a standalone treatment option for the treatment of established nonunions and delayed unions as it is believed to promote healing in all phases of fracture healing viz., inflammatory, intramembranous ossification, chondrogenesis, endochondral ossification and remodelling. In the current scenario of varying results and unclear clinical role of LIPUS therapy, we present a prospective case series of fracture nonunions and delayed unions treated with LIPUS therapy at a large District General Hospital.

## Introduction

The incidence of nonunion of fractures varies between 5-10% and has been steadily rising owing to improved life expectancy following severe injuries [1]. The incidence of nonunion is also directly related to the severity of causative injury with multiply injured patients being at a higher risk of developing long-term orthopaedic complications such as nonunion [2-3]. Once a nonunion is established, the chances of spontaneous healing are deemed to be quite low [4]. Fracture nonunion and delayed unions continue to be a challenge in clinical practice with nonunions having a considerable impact on patient’s quality of life causing functional and psychosocial disability along with it being the cause of an economic burden owing to prolonged disability and downtime of job [5]. Although ultrasound is routinely indicated as a diagnostic modality in Trauma & Orthopaedics, its use as a therapeutic intervention is still not well understood [6].

The biological use of ultrasound was first explored by Wood and Lumis back in 1924 wherein they described the biological changes related to ultrasound treatment [7]. The first evidence of the use of ultrasound in the treatment of fracture nonunions however can be traced to 1983 wherein success rates of almost 70% were reported following the use of ultrasound therapy in established nonunions [8]. The high healing rates followed by a slew of successful clinical trials done in the United States (US) led to the approval of Low-Intensity Pulsed Ultrasound (LIPUS) therapy by the US Food and Drug Administration in 1994 [9]. The approval was initially granted for accelerating the healing of certain fresh fractures but was later extended to encompass established nonunions in 2000 [9]. The approved ultrasound signal for LIPUS is the 1.5 MHz sinusoidal wave modulated in bursts of 200 ms at a repetition frequency of 1 kHz. Recent literature has projected LIPUS as a safe and effective home treatment option for delayed unions and nonunions with success rates of up to 93% [10]. LIPUS is being widely recommended as a standalone treatment option rather than an adjuvant for the treatment of established nonunions and delayed unions as it is believed to promote healing in all phases of fracture healing viz., inflammatory, intramembranous ossification, chondrogenesis, endochondral ossification, and remodelling [9]. Moreover, LIPUS is also being widely considered as a viable treatment option for nonunions and delayed unions in individuals who are not ideal candidates for surgery [11]. This includes patients who are suffering from dementia, old age, multiple organ failure, and coma [11]. In healthy individuals, however, Rutten et al. found that LIPUS was effective in reducing the mean time to the radiographic union by 39.8 days [12]. Zura et al. in their large observational cohort study of 767 patients reported a radiographic union rate of 80% in established nonunions where LIPUS was used as a standalone treatment modality without any adjuncts [13].

In the current scenario of varying results and unclear clinical role of LIPUS, we present a prospective case series of fracture nonunions and delayed unions treated with Low-Intensity Pulsed Ultrasound (LIPUS) therapy at a District General Hospital.

## Materials and methods

The LIPUS therapy has been in use for delayed unions and nonunions at our institute since 2016. A Trust pathway detailing indications and referral criteria is in place, and this was circulated amongst the members of the staff again.

Inclusion criteria

All patients having reached the age of 16 or above with established nonunions or delayed unions were included in the study. Patients who had not attained the age of 16 were not included as the role of LIPUS in nonunions or delayed unions of the immature skeleton is not well supported by the current body of literature [4]. A minimum period of six months post-surgery/post-injury was allowed for the fractures to heal, before being considered for LIPUS therapy. Only patients who were willing to accept LIPUS therapy as a standalone treatment option were included.

Exclusion criteria

Patients with unstable fractures or patients who were having a concomitant infection were excluded from the study. All patients with spinal fractures or skull fractures were also excluded. The patients who were not willing to accept the use of LIPUS therapy as a standalone treatment option were excluded. Patients in whom wound care or current wound status hindered ultrasound-skin contact were not included.

Methods

All prospective patients were seen face to face in the outpatient clinic for a clinical evaluation. Plain radiograph with standard Anteroposterior (AP) and Lateral views were obtained to establish the correct diagnosis. Nonunion was confirmed by the absence of any cortical or cancellous bone bridge between the fracture fragments. In instances where bone bridging was unclear on X-rays or there was a presence of bulky osteosynthesis material hindering appropriate diagnosis, Computed Tomography (CT) scan was obtained to establish the diagnosis. The patients were explained about the role of LIPUS, its proposed mechanism of action, and its principle. Once the patients agreed to the inclusion, they were provided with the portable LIPUS device, namely EXOGEN® (Bioventus LLC, London, United Kingdom) and treatment commenced. The patients were provided with training to correctly use the device at home. The training was delivered by the prescribing physician and a representative from the LIPUS device manufacturer's company. Leaflets with detailed instructions on how to use the device were also provided to the patients. Contact numbers of the relevant team members were given to the patients, in case of any queries. 

The patients were explained that the treatment would require them to use the LIPUS device for 20 minutes every day. They were told that the device would need to be centred over the site of nonunion or delayed union. The target site for the LIPUS device was marked for reference on the affected site after an AP X-ray of the non-united fracture was taken with a radio-opaque mark on the skin corresponding to the fracture site. This was made on an easily accessible part of the affected site, for example, the anterior aspect of a limb. Visual Analog Scale (VAS) was also utilized to assess pain levels of the patients on commencement of the treatment and end of the treatment. The patients were followed up every three months for a minimum period of six months. Earlier appointments were also made at the request of patients. The maximum period for treatment with LIPUS was agreed to be one year. The patients were instructed to follow the LIPUS device instructions with at least 90% adherence to be deemed compliant with treatment.

A total of 49 patients were enrolled from December 2016 to June 2020 (42 months). The relationship between the radiographic union and LIPUS was analysed. The effect of fracture healing on the VAS score was also analysed. The case series was conducted with the aim to understand the efficacy of LIPUS therapy on fracture nonunions and delayed unions. It sought to assess the results of LIPUS therapy on nonunions and delayed unions along with the limitations of the therapy.

## Results

The group of 49 patients enrolled between December 2016 to June 2020 comprised 16 males and 33 females aged from 24 years to 86 years (Table 1), with an average age of 54.61 years. All the enrolled patients were followed up for a minimum period of six months. All the patients had upper or lower extremity fractures with no patients with spinal or skull fractures recruited onto the study. The average time gap in the referral of patients from the date of injury to the first LIPUS therapy clinic appointment was 9.2 months (range 6-18 months).

**Table 1:** Summary of the patients recruited ORIF - Open Reduction and Internal Fixation; Exogen - Low-intensity pulsed ultrasound (LIPUS) device used for the therapy

Age of Patient	Diagnosis	Exogen Duration	Result
60 years	Nonunion Distal Radius	6 months	Union
70 years	Delayed Union post Midshaft Humerus ORIF	6 months	Union
70 years	Nonunion post Distal Femur Fracture ORIF	12 months	Non-union
83 years	Delayed Union post Distal Radius ORIF	12 months	Non-union
31 years	Delayed Union Tibial Shaft Fracture	6 months	Union
45 years	Nonunion Tibial Shaft Fracture	12 months	Union
59 years	Nonunion 5th Metatarsal Fracture	Lost to follow up	-
86 years	Nonunion post Midshaft Femur Fracture ORIF	12 months	Non-union
50 years	Nonunion Post Distal Femur ORIF	6 months	Union
53 years	Nonunion post 5th Metatarsal Fracture	6 months	Union
31 years	Nonunion post Scaphoid Fracture	6 months	Union
40 years	Delayed union post Distal Tibia Fracture	6months	Union
70 years	Nonunion post Talus Fracture ORIF	12 months	Non-union
42 years	Delayed union post 5th Metatarsal Fracture	3 months	Union
60 years	Nonunion post Olecranon Fracture ORIF	3 Months	Union
68 years	Nonunion Midshaft Humerus Fracture	12 months	Non-union
44 years	Delayed union 5th Metatarsal Fracture	Lost to follow up	-
40 years	Delayed union post Tibial Shaft Fracture	6 months	Union
51 years	Nonunion post 5th metatarsal ORIF	4 months	Union
56 years	Nonunion post 5th Metatarsal fracture	4 months	Union
54 years	Delayed union post Distal Radius Fracture ORIF	3 months	Union
60 years	Nonunion post Ankle Weber B Fracture	6 months	Union
45 years	Nonunion post Midshaft Femur Fracture ORIF	6 months	Union
49 years	Nonunion post Midhsaft Femur Fracture ORIF	6 months	Union
36 years	Delayed union 5th Metatarsal Fracture	3 Months	Union
24 years	Delayed union 3rd Metatarsal + Cuneiform Fracture	5 months	Union
63 years	Delayed union 5th Metatarsal fracture	5months	Union
47 years	Nonunion post 1st Metatarsal ORIF	6 months	Union
66 years	Delayed union Clavicle Fracture	3 months	Union
55 years	Delayed union calcaneum Fracture	3 months	Union
53 years	Nonunion post Midshaft Humerus Fracture ORIF	12 months	Non-union
73 years	Nonunion post 5th Metatarsal Fracture	6 months	Union
56 years	Nonunion post Ankle Weber B Fracture	4 months	Union
77 years	Delayed union Post Distal Radius Fracture ORIF	3 months	Union
26 years	Delayed union post Ankle Fracture ORIF (Weber B)	5 months	Union
55 years	Delayed union 5th Metatarsal Fracture	5 months	Union
54 years	Nonunion post Proximal Tibia Fracture ORIF	12 months	Non-union
29 years	Nonunion post 5th Metatarsal Fracture ORIF	3 months	Union
53 years	Nonunion post 1st Metatarsal Fracture ORIF	Lost to Follow up	-
43 years	Delayed union post 2nd, 3rd, 4th Metatarsal Fracture ORIF	6 months	Union
73 years	Delayed union Ankle Weber B Fracture	Lost to follow up	-
59 years	Nonunion post Distal Radius Fracture ORIF	6 months	Union
76 years	Nonunion post Proximal Tibia Fracture ORIF	Noncompliant	-
61 years	Nonunion post Midshaft Humerus Fracture ORIF	Noncompliant	-
70 years	Nonunion post Talus Fracture ORIF	12 months	Non-union
58 years	Nonunion post Ankle ORIF (Weber B)	12 months	Non-Union
44 years	Delayed union post Midshaft Tibia Fracture ORIF	5 months	Union
46 years	Nonunion post 1st Metatarsal Fracture ORIF	Noncompliant	-
62 years	Delayed union post Ankle Fracture ORIF (Weber B)	6 months	Union

Four patients were lost to follow up and three others were deemed to be noncompliant. The required threshold of 90% adherence was met by the remaining 42 patients. We noted mean compliance of 95% (range 91 - 97%). In this cohort of 42 compliant patients, 18 patients were managed conservatively upon initial presentation to fracture clinic, while the remaining 24 patients underwent surgery as the first line of management.

**Figure 1:**
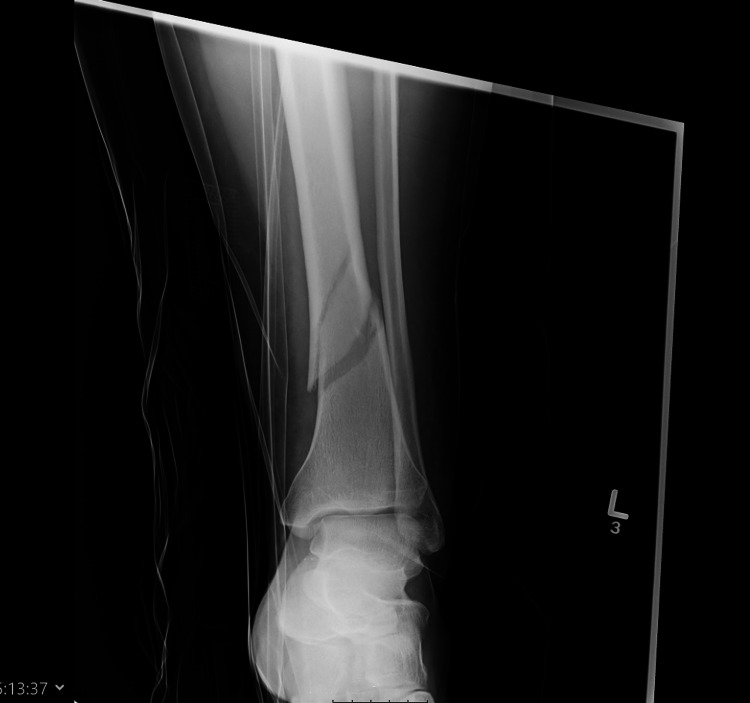
Distal Tibia fracture at the time of injury (AP View) AP - Anteroposterior

**Figure 2:**
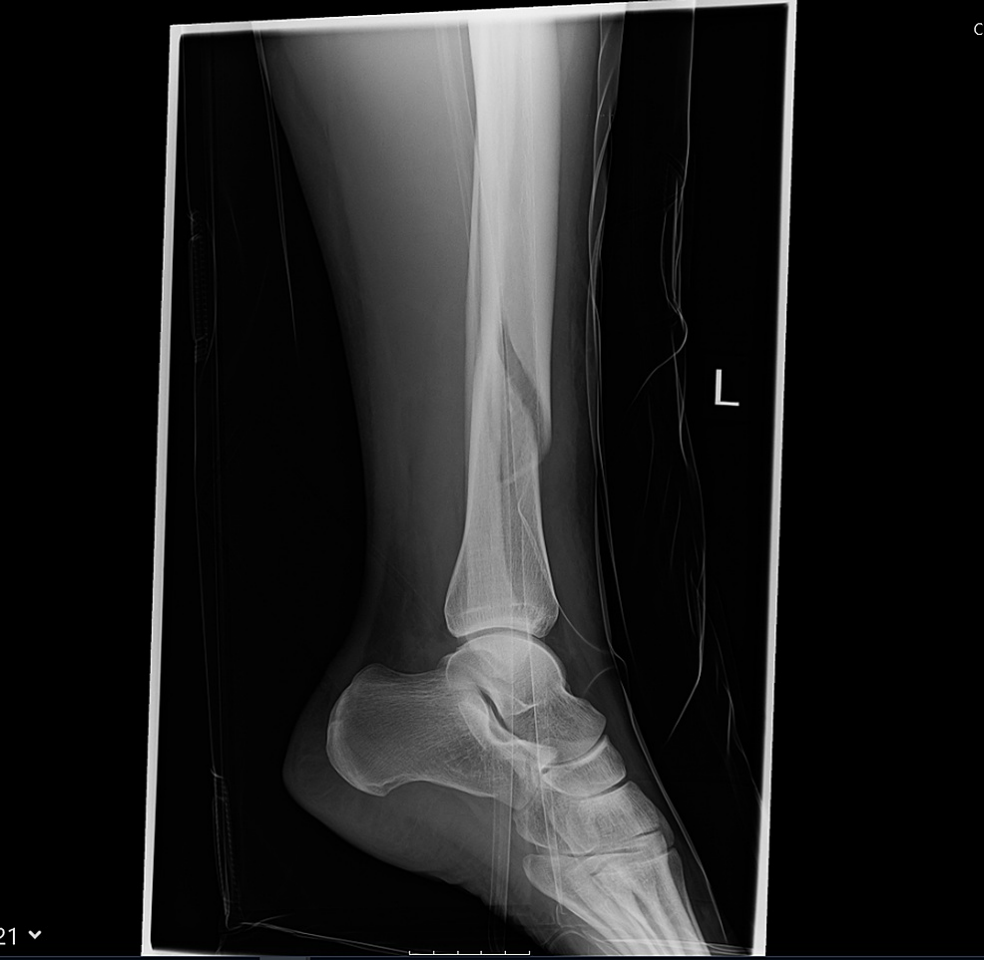
Distal Tibia fracture at the time of injury (Lateral view)

**Figure 3:**
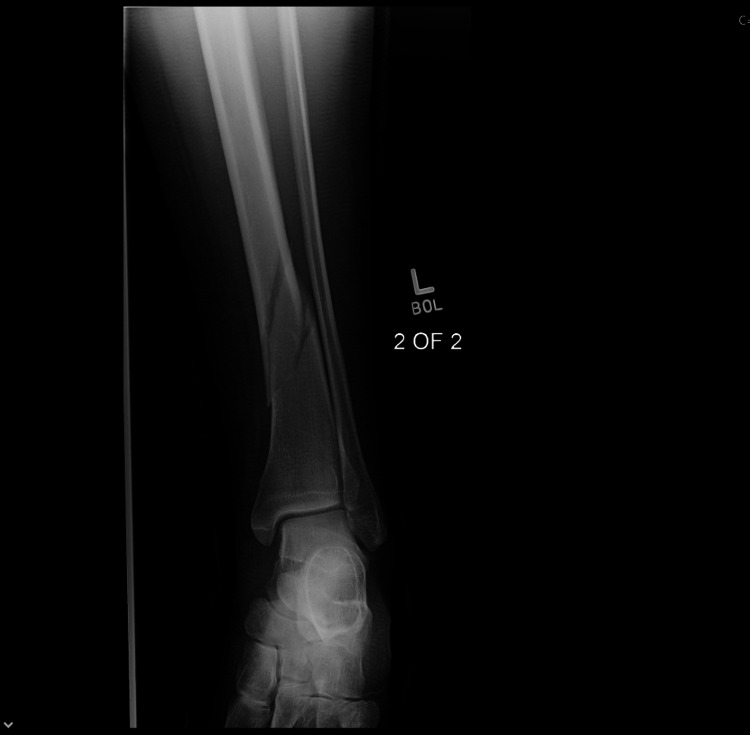
Distal Tibia fracture at the time of initiation of LIPUS therapy (6 months post injury - AP view) AP - Anteroposterior; LIPUS - Low-Intensity Pulsed Ultrasound

**Figure 4:**
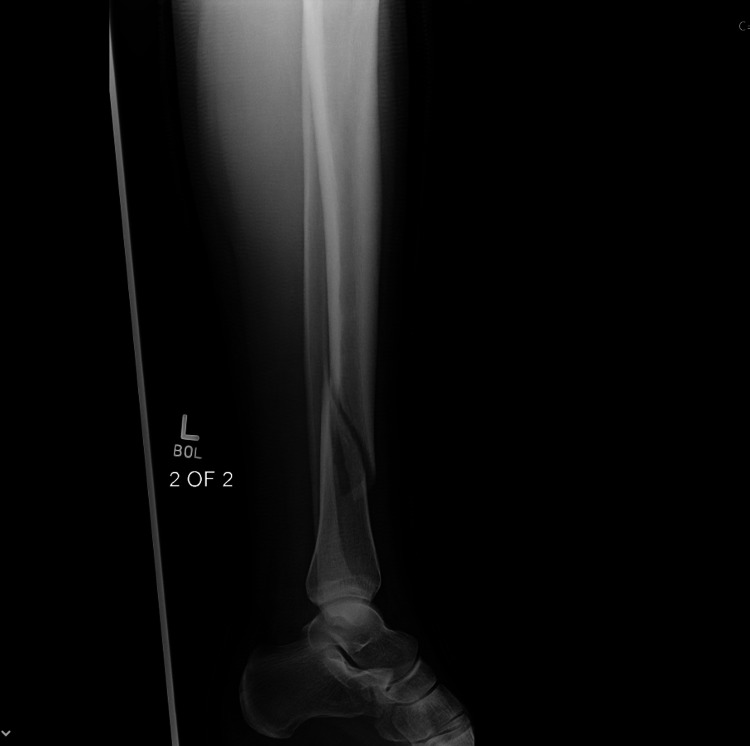
Distal Tibia fracture at the time of initiation of LIPUS therapy (6 months post injury - lateral view) LIPUS - Low-Intensity Pulsed Ultrasound

**Figure 5:**
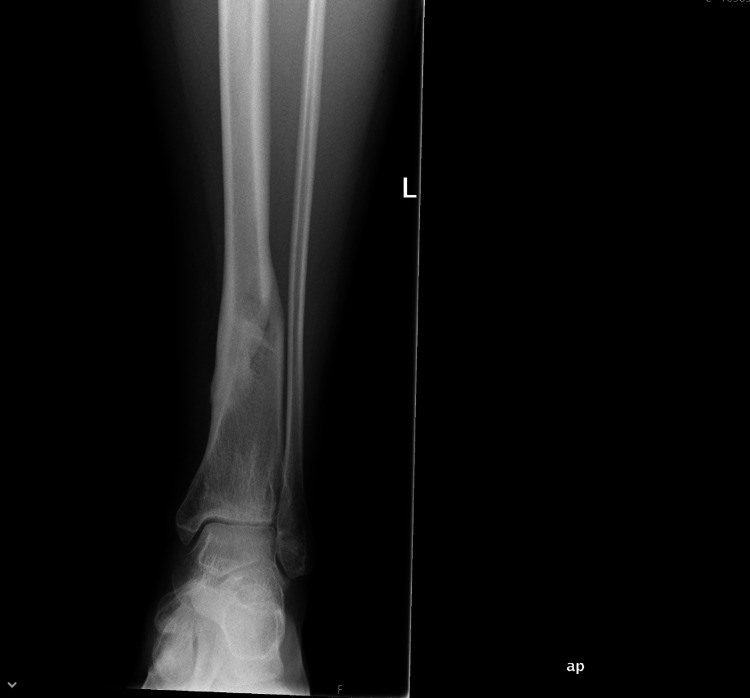
Union status at the completion of 6 months of LIPUS therapy (AP View) AP - Anteroposterior; LIPUS - Low-Intensity Pulsed Ultrasound

**Figure 6:**
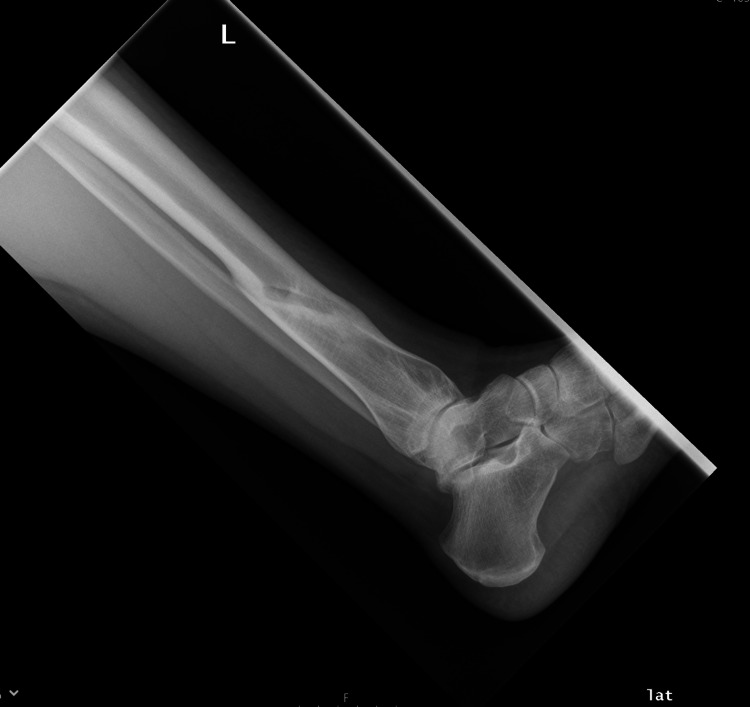
Union status at the completion of 6 months of LIPUS therapy (Lateral view) LIPUS - Low-Intensity Pulsed Ultrasound

Thirty-three patients out of 42 showed a radiographic union on X-rays at the end of LIPUS therapy treatment; for example, see Figures 1-6. This is indicative of the 78.57% success rate of LIPUS in the treatment of nonunions. In the nine failed patients, there was no evidence of union despite being given the LIPUS treatment for the maximum pre-agreed period of one year. Out of these nine patients, one patient was post midshaft Humerus Open Reduction and Internal Fixation (ORIF), one patient post midshaft humerus fracture managed conservatively, one post Distal Femur ORIF, one post Distal Radius ORIF, one post midshaft Femur ORIF, one post Ankle ORIF, two post Talus ORIF and one post Proximal Tibia ORIF.

The pre-LIPUS therapy mean VAS score was found to be 5.1 (range 3-7). This improved to 3.9 (range 0-6) upon culmination of LIPUS therapy in the 42 compliant patients. The nonunions associated with fractures that failed to unite comprised both atrophic and hypertrophic nonunion. The nonunion post Proximal Tibia ORIF, midshaft Femur ORIF, and Distal Femur ORIF was of the hypertrophic type, while the remaining were atrophic type. The maximum fracture site gap in the non-united fractures was found to be not less than 9mm (range 9-10mm) at the commencement of LIPUS therapy. This may suggest that a fracture site gap less than 9mm has a positive role in predicting the success of LIPUS therapy.

## Discussion

Low-intensity pulsed ultrasound has been drawing increased attention globally owing to its therapeutic benefits while avoiding any significant risks like surface temperature change caused by the use of high-intensity ultrasound in humans [14]. National Institute for Health and Care Excellence (NICE) in the United Kingdom (UK) has also supported the use of LIPUS therapy in nonunions and delayed unions under controlled settings while suggesting further research to study its mechanism of action, principle, and efficacy [15]. A multi-centre randomised controlled trial (RCT) conducted in Germany reported better healing in patients who had LIPUS therapy by a margin of 34% as compared to the control or sham group [16]. Given the fact that established nonunions show no signs of spontaneous healing, the findings of LIPUS therapy in various studies around the world are compelling. Bashardoust et al. in a meta-analysis reported that patients who received LIPUS therapy exhibited accelerated radiographic union in 14 out of the 20 studies reviewed [17]. Bawale et al. have also compared success rates of LIPUS therapy to success rates of surgical management of nonunions, with both having reportedly similar success rate of around 70% [4]. This also opens up the debate of whether attempting surgical management of an established nonunion is a better option than trying LIPUS therapy provided both have similar success rates and LIPUS therapy may comparatively be much safer and sometimes even faster. Jingushi et al. suggested a significant relationship between union rate and the period from the most recent operation [18]. They have suggested that LIPUS therapy be initiated within six months of the most recent operation in all cases of post-operative nonunion and delayed union owing to the high efficacy, safety, and tolerability of the therapy. The cost of LIPUS therapy is also estimated to be almost 49% less than the cost of surgical revision in the National Health Services (NHS) of the United Kingdom [15]. Thus, LIPUS therapy can also be projected as an economic and viable alternative to revision surgery provided it is proposed to have similar success rates as revision surgery. It has been projected that LIPUS therapy can save almost £2200 per patient who does not undergo revision surgery. In a resource stretched and heavily burdened NHS, this may well prove to be a very cost-effective alternative to surgeries.

Although it has been widely published that LIPUS therapy may be effective in reducing time to radiographic union, its role in actually providing a beneficial or positive effect through accelerated functional recovery is still unclear. Promoting radiographic union may not directly correlate to improved functional levels or clinical healing. Hence, although the role of LIPUS in promoting radiographic union may be explained to some extent, the role of LIPUS in the clinical healing of nonunions and delayed unions is still debatable and not well understood [12]. Jiang et al. in their systematic review found that LIPUS therapy had a positive effect on chronic delayed unions and nonunions which had not healed for almost 10 years, but were still unable to comment on the role of LIPUS therapy in clinical healing [14]. Thus, the role of LIPUS therapy in clinical healing needs to be examined further. 

## Conclusions

Our series demonstrates a success rate of 78.57% with the use of LIPUS therapy in the treatment of nonunion or delayed union. This is in line with the success rates that have been reported in the current literature. The LIPUS therapy union rate is also comparable to the union rates obtained following surgical revisions of nonunions and delayed unions. As outcomes are comparable, one may consider LIPUS therapy over surgical treatment since safety favours LIPUS therapy owing to its non-invasiveness. However, large multi-centre studies may be needed to confirm our findings before large scale adoption of LIPUS therapy can be recommended. The main limitation of our study is that it is a single-centre clinical series. Multi-centre studies and randomised controlled trials may be needed to study the efficacy and indications of LIPUS therapy in detail. Although, current evidence supports the use of LIPUS therapy to promote radiographic union of delayed unions and nonunions, further studies are required to delineate the exact role of LIPUS therapy in clinical healing along with further studies to understand the mechanism of action of LIPUS therapy in nonunions and delayed unions.
